# Supplementary material for: Psychedelic 5-HT2A agonist increases spontaneous and evoked 5-Hz oscillations in visual and retrosplenial cortex
Source: Commun Biol. 2026 Jan 12;9:216. doi: 10.1038/s42003-025-09492-9 (PMC12894671; doi:10.1038/s42003-025-09492-9)
Supplement: Supplementary file 2 — Supplementary information [file 42003_2025_9492_MOESM2_ESM.pdf]

Supplementary information

Psychedelic 5-HT2A agonist increases spontaneous and evoked 5-Hz oscillations in visual and retrosplenial cortex

Callum M. White[1,2*], Zohre Azimi[1*], Robert Staadt[1*], Chenchen Song[3], Thomas Knöpfel[4**+], Dirk Jancke[1,2**+]

[1] Optical Imaging Group, Institut für Neuroinformatik, Ruhr University Bochum, Germany

[2] Monoaminergic Neuronal Networks & Diseases (MoNN&Di), Ruhr University Bochum, Germany

[3] Lee Kong Chian School of Medicine, Nanyang Technological University, Singapore

[4] JC STEM Laboratory for Neuronal Circuit Dynamics, Hong Kong Baptist University, Hong Kong SAR

* These authors contributed equally

** These authors contributed equally

[+]**Correspondence:** Dirk Jancke, PhD, dirk.jancke@rub.de, Thomas Knöpfel, PhD, tknopfel@hkbu.edu.hk

Supplementary information

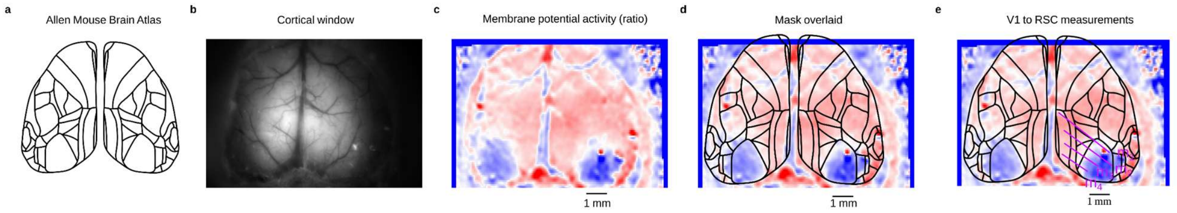

Supplementary Figure 1: Identification of wide-field cortical regions. a: Regions of the cortical surface was recreated using the Allen Mouse Brain Atlas (brain-map.org). **b**: An example of the cortical window field of view. **c**: The mean (N=30 trials) hyperpolarisation (identified using the local minima following visually evoked depolarisation) of the membrane potential activity within the cortical window of **b** following a visual stimulus (100% horizontal moving grating for 0.2s). **d**: Manually aligned cortical regions shown in **a** to the regions to the depolarisation in V1, then scaled to the experimental magnification factor. **e**: Estimated distances from V1 to RSC (m1 = 3.166 mm, m2 = 2.884 mm, m3 = 1.968 mm, m4 = 1.486 mm), measured using optical imaging with a calibrated millimeter scale (measurement calibration performed in ImageJ).

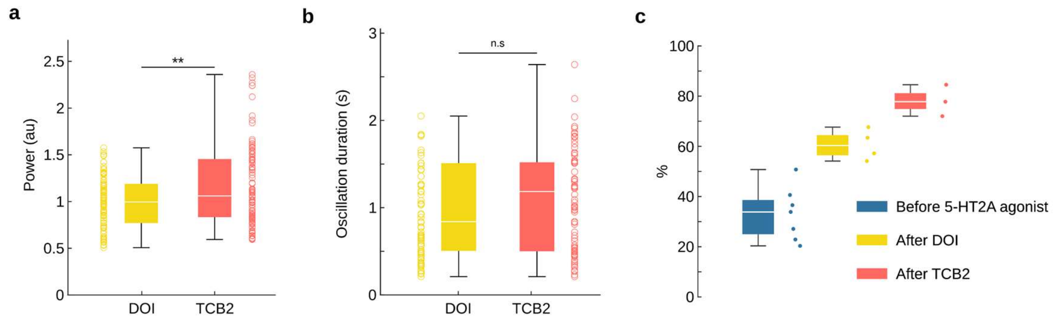

Supplementary Figure 2: Drug dependency of evoked oscillations in V1. a: Power of oscillations after treatment of DOI (yellow) and TCB2 (red), comparing the individual trials (**, p-value=1.09e-03, Welch's t-test). Data based upon N = 7 experiments in 5 mice; N_{DOI}=4; N_{TCB2}=3). **b**: same as ***a***, for duration of oscillations across individual trials (n.s, p-value=0.0796, Welch's t-test). **c:** TCB2 induced a more pronounced effect in the probability of 5-Hz oscillations occurring in V1 (oscillations detected using FFT analysis). Boxplots follow Tukey's method: median (middle line), 25th, 75th percentile (box), ± 2.7 sigma (whiskers).

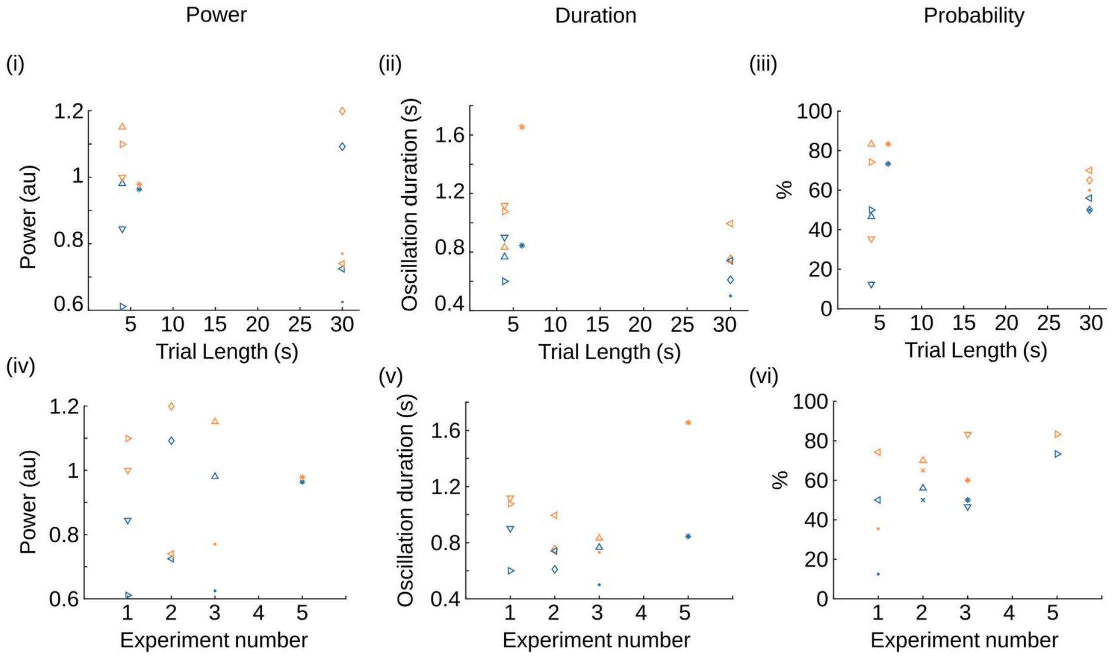

Supplementary Figure 3: Independence of modulation for evoked 5-Hz oscillations in V1. i-iii, shows the independence of power, duration and probability against trial length. **iv-vi**, same as **i-iii** for the number of similar experiments the mouse had carried out at each data point.

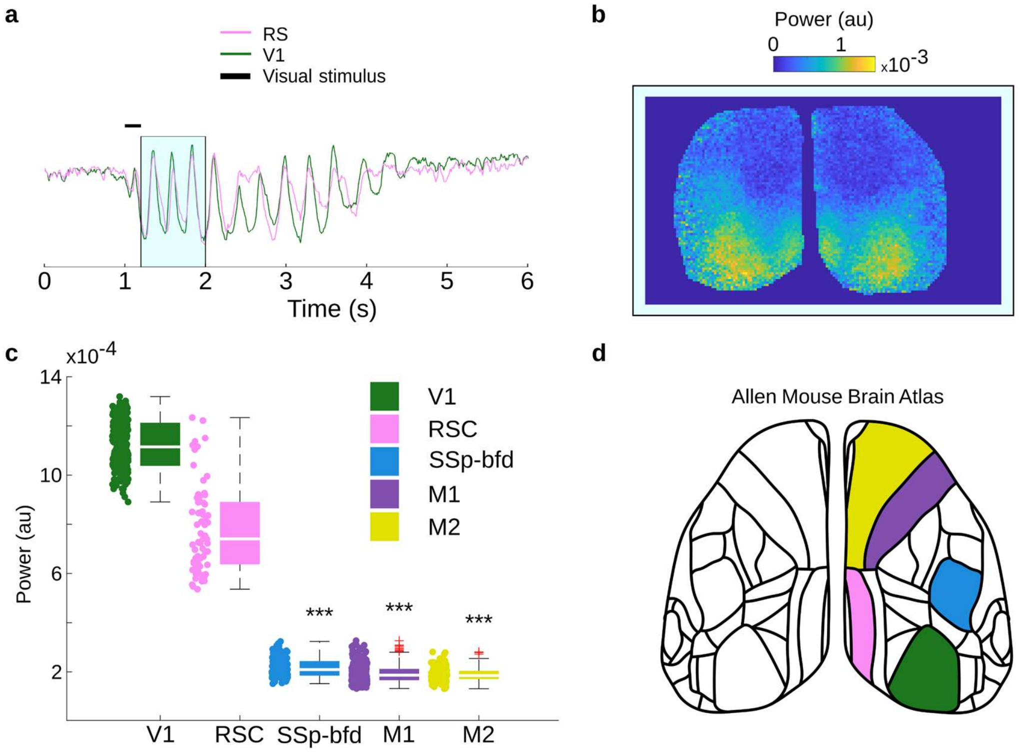

Supplementary Figure 4: Distribution of visually-evoked 5-Hz power across the mouse cortex. a: The voltage activity across V1 and RSC of a single trial with a visually evoked 5-Hz oscillation. **b**: Spectral analysis of each pixel during the oscillation in a (blue shaded region), showing the mean 5-Hz power. **c**: The average spectral power for each pixel (mean over all evoked oscillation trials before and after 5-HT2A agonist injection, n=3 mice and N=175 trials) within a post-stimulus time window for each cortical area. Boxplot follows Tukey's method: median (middle line), 25th, 75th percentile (box), ± 2.7 sigma (whiskers), outliers are plotted with a red '+'. **d**: Regions of the cortical surface (the Allen Mouse Brain Atlas, brain-map.org) as shown in **Supplementary Fig. 1**, labelling V1, Primary visual cortex (green); RSC, Retrosplenial cortex (pink); SSp-bfd, Primary somatosensory area- barrel field (blue); M1, Primary motor area (purple); M2, Secondary motor area (yellow). (**Statistics**: V1 – SSp-bfd, ***, 4.73e-263; V1 – M1, ***, 5.62e-252; V1 – M2, ***, 6.82e-240; RSC – SSp-bfd, ***, 1.74e-37; RSC – M1, ***, 3.53e-38; RSC – M2, ***, 2.48e-38. All conducted using a Welch's t-test).

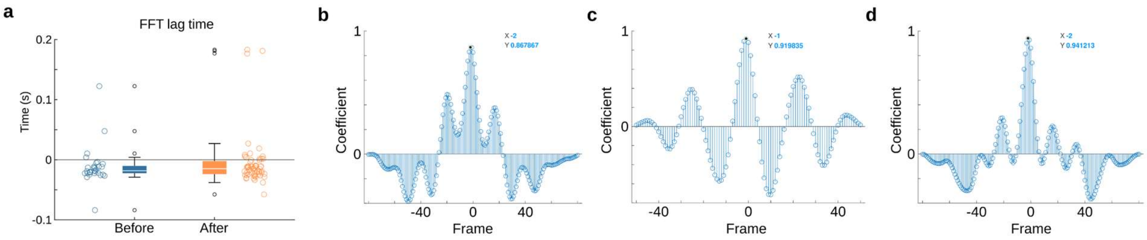

Supplementary Figure 5: Secondary method of calculating lag time of 5-Hz oscillations between V1 and RSC with single trial examples of cross correlation. a: The lag time between signals in V1 and RSC across single trials, using FFT analysis to calculate the phase difference and subsequently the lag time. Showing a median lag of -18.22ms (before, blue) and -14.85ms (after, orange). **b-d**: Single trial examples of the cross correlation found between signals in V1 and the RSC, with a coefficient of 0.87 (**b**, lag of -2), 0.92 (**c**, lag of -1) and 0.94 (**d**, lag of -2).

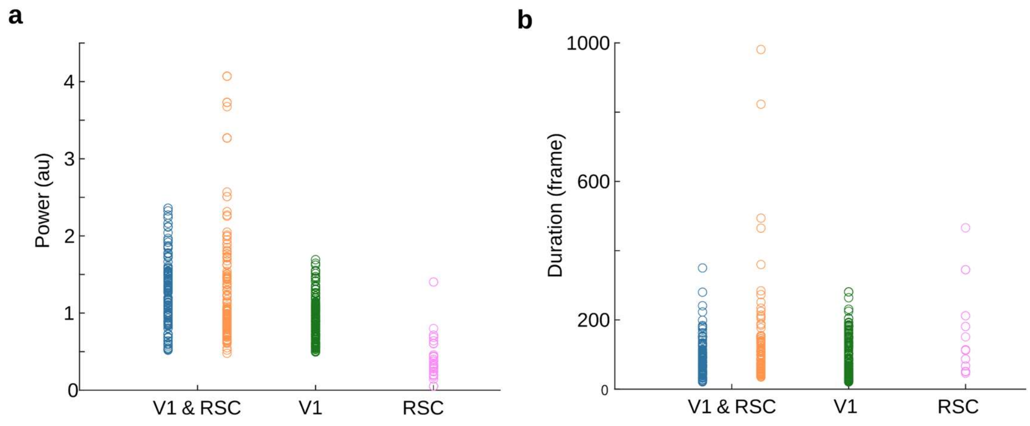

Supplementary Figure 6: Power and duration for region specific evoked oscillations. a: The power for evoked oscillations co-occurring in V1 and RSC, V1 only and RSC only. **b**: same as **a**, for duration of evoked oscillations. One image frame = 10ms.

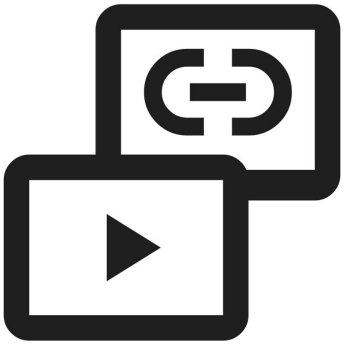

Supplementary video 1: Visualisation of the evoked 5-Hz oscillation dynamics in V1 and RSC. Mean activity across trials (N=30, with and without oscillations) based on the experiment shown in **Fig. 3a-b**, before (left) and after (right) injection of a 5-HT2A agonist. Above, showing the spatiotemporal dynamics of the cortex-wide voltage activity (regions outlined using the Allen Mouse Brain Atlas, brain-map.org) and the spatial mean (below) across V1 (green) and RSC (pink). (video file: Supplementary_video_1_v2_1080p.mp4)
