# Supplementary material for: Psychedelic 5-HT2A agonist increases spontaneous and evoked 5-Hz oscillations in visual and retrosplenial cortex
Source: Commun Biol. 2026 Jan 12;9:216. doi: 10.1038/s42003-025-09492-9 (PMC12894671; doi:10.1038/s42003-025-09492-9)
Supplement: Supplementary file 3 — Description of Additional Supplementary Files [file 42003_2025_9492_MOESM3_ESM.pdf]

Description of Additional Supplementary File

File name: Supplementary Data

Description: Numerical source data for all graphs

File name: Supplementary video 1
Description: Visualisation of the evoked 5-Hz oscillation dynamics in V1 and RSC
